# Comparison of stemless and conventional stemmed shoulder arthroplasties in shoulder arthropathy: A meta-analysis

**DOI:** 10.1097/MD.0000000000023989

**Published:** 2021-02-12

**Authors:** Young-Soo Shin, Woo-Seung Lee, Jun-Sung Won

**Affiliations:** a Department of Orthopaedic Surgery, Veterans Health Service Medical Center, Seoul; b Department of Orthopedic Surgery, Hallym University School of Medicine, Chucheon, Republic of Korea.

**Keywords:** anatomical shoulder arthroplasty, complication rates, meta-analysis, reverse shoulder arthroplasty, stemless

## Abstract

**Background:**

It is unclear whether stemless shoulder prosthesis lead to better clinical outcomes than conventional stemmed shoulder prosthesis. The purpose is to compare clinical outcomes and complication rates after surgery in patients with shoulder arthropathy treated with stemless or conventional stemmed shoulder prosthesis.

**Method:**

All studies comparing the constant score (CS), range of motion (ROM), and complication rates after surgery in patients with shoulder arthropathy treated with stemless or conventional stemmed shoulder prosthesis were included. The major databases MEDLINE, EMBASE, the Cochrane Library, Web of Science, and SCOPUS were searched for appropriate studies from the earliest available date of indexing through March 31, 2019. No restrictions were placed on language of publication.

**Results:**

A total of 6 studies met the inclusion criteria and were analyzed in detail. Overall postoperative ROM (95% CI: 3.27 to 11.92; *P* < .01) was significantly greater for stemless prosthesis compared to conventional stemmed prosthesis. However, postoperative CS (95% CI: −2.98 to 7.13; *P* = .42) and complication rates (OR 1.22, 95% CI: 0.48–3.08; *P* = .68) were did not differ significantly between the 2 groups.

**Conclusion:**

This meta-analysis revealed that postoperative CS and complication rates did not differ significantly between the 2 treatment methods, stemless shoulder prosthesis and conventional stemmed shoulder prosthesis, for shoulder arthropathy. However, stemless shoulder prosthesis resulted in better outcomes than conventional stemmed shoulder prosthesis in terms of postoperative ROM.

**Level of evidence:**

Level III, Therapeutic study.

## 1 Introduction

Shoulder arthroplasty is a well-established treatment for severe osteoarthritis of the glenohumeral joint to relieve pain and to restore shoulder function. When a long-stem humeral component is implemented, extensive bone ingrowth or use of a cemented long-stem prosthesis make it difficult to remove the fixed components during revision shoulder arthroplasty. Moreover, as the traditional fixation method is difficult to apply in patients with degenerative changes of the proximal humerus, these patients often require additional procedures such as osteotomy of the greater tuberosity of the proximal humerus.^[1]^ After the insertion of the long-stem prosthesis, various complications could develop, including bone resorption around the proximal part of the bone caused by stress shielding,^[2–4]^ osteolysis due to polyethylene residues, and periprosthetic fracture.^[5,6]^ Periprosthetic humerus fractures account for approximately 20% of all complications associated with shoulder arthroplasty.^[7–10]^ A short-stem humeral component was introduced to reduce the risk of complications caused by the long-stem prosthesis.^[11–14]^ The long-stem prosthesis was designed to hold the implant by applying pressure to the humeral shaft, whereas the short-stem prosthesis was designed to hold the implant by compressing the cancellous bone of the proximal metaphysis. Therefore, the short-stem prosthesis can reduce the stress shielding of the proximal part of the humerus, unlike the long-stem prosthesis. Most recently, stemless shoulder arthroplasty was newly introduced to minimize the risk of stem-associated complications by fixing the prosthesis on the metaphysis of the humeral neck. Stemless shoulder arthroplasty not only allows for an easier revision shoulder arthroplasty but also provides further support for the maintenance of the bone quality of the proximal humerus.^[15]^ However, there are concerns with stemless shoulder prosthesis; they cannot be used in cases with poor bone quality, especially in elderly patients, and the risk of intraoperative fractures of the greater tuberosity by a tight press-fit metaphyseal central anchor or screw fixation.^[16]^ Although many studies have reported the clinical outcome and complication rates of patients who underwent shoulder arthroplasty with 1 of the 2 prosthesis, few comparative studies exist. This meta-analysis was performed to assess clinical outcomes and complication rates after surgery in patients with shoulder arthropathy treated with stemless or conventional stemmed shoulder implants. The hypothesized is that stemless shoulder implants would lead to better clinical outcomes and lower complication rates than conventional stemmed shoulder implants in patients with shoulder arthroplasty at final follow-up.

## 2 Materials and methods

### 2.1 Data and literature sources

Although the current study involved human participants, ethical approval and informed consent from participants were not required because all data were acquired from previously published studies and analyzed anonymously without any potential harm to participants. The comprehensive databases of MEDLINE (January 1, 1976–March 31, 2019), EMBASE (January 1, 1985–March 31, 2019), Web of Science (January 1, 1980–March 31, 2019), SCOPUS (January 1, 1980–March 31, 2019), and the Cochrane Library (January 1, 1987–March 31, 2019), were searched for studies that compared Constant Score (CS), range of motion (ROM), and complications in patients treated with stemless or conventional stemmed shoulder implants with short-term (<5 years) follow-up. There were no restrictions on language. Search terms used in the title, abstract, MeSH, and keywords fields were (“shoulder joint” [MeSH] OR “arthroplasty” [MeSH] OR “shoulder prosthesis” [MeSH] OR “replacement” [MeSH]) AND “stemmed” [tiab] OR “stemless” [tiab]OR “stem” [tiab] OR “stems” [tiab]OR “shoulder arthroplasty” [tiab] OR “shoulder prosthesis” [tiab] OR “artificial shoulder joint” [tiab] OR “shoulder implant” [tiab] OR “shoulder replacement” [tiab]). After the initial electronic search, additional relevant articles and bibliographies from identified studies were hand searched through other sources, including abstracts from annual meetings of the American Academy of Orthopedic Surgeons (AAOS) and the Osteoarthritis Research Society International (OARSI). We also searched weekly downloads of “Arthroplasty” articles in 6 journals (American Journal of Orthopedics; Archives of Orthopedic and Trauma Surgery; Journal of Arthroplasty; Journal of Bone and Joint Surgery American volume; Journal of Bone and Joint Surgery British volume; Orthopedics). The search was performed independently by 2 reviewers.

### 2.2 Study selection

Two reviewers independently selected relevant studies for full review by searching through titles and abstracts. The full text copy of each article was reviewed if the abstract did not provide enough data to make a decision. Studies were included in the meta-analysis if they

1. assessed postoperative CS, ROM, and complication rates of patients with shoulder arthropathy treated with stemless or conventional stemmed shoulder prosthesis;
2. had mean follow-up duration of 6 months or longer;
3. simultaneously reported direct comparisons of stemless or conventional stemmed shoulder prosthesis in studies published after 2000, to avoid out-of-date prosthetic models;
4. included basic data on at least 1 of the following 3 parameters: postoperative CS, ROM, or complication rates;
5. reported the number of subjects in each group and the means and standard deviations for the 3 parameters, and
6. used adequate statistical methods to compare parameters between groups.

Studies were excluded if they

1. had missing or inadequate outcome data, such as standard deviations or ranges of values;
2. were case reports, expert opinions, reviews, commentaries, or editorials;
3. were abstracts only;
4. focused on animal in vivo or human in vitro work.

### 2.3 Data extraction and assessment of methodological quality

Two reviewers independently recorded data from each study using a predefined data extraction form and resolved any differences by discussion. Recorded variables were those associated with surgical outcomes, such as postoperative CS, ROM, and complication rates, for patients with either stemless or conventional stemmed shoulder prosthesis. Sample size and the means and standard deviations of surgical outcomes in each group were also recorded. Two reviewers independently assessed the methodological quality of the studies. For prospective RCTs, methodological quality was assessed with the modified Jadad scale, which assesses randomization, blinding, withdrawals and dropouts, inclusion and exclusion criteria, adverse reactions, and statistical analysis. High quality studies have scores of 4–8, whereas low quality studies have scores of 0–3.^[17]^ For the Newcastle-Ottawa Scale, as recommended by the Cochrane Non-Randomized Studies Methods Working Group, we assessed studies based on 3 criteria: selection of the study groups, comparability of the groups, and ascertainment of either the exposure or the outcome of interest for case-control and cohort studies. The maximum score observed was 9 points, and total scores lower than 4 points were considered low in quality. Two reviewers resolved all differences by discussion, and their decisions were subsequently reviewed by a third investigator.

### 2.4 Data synthesis and analysis

The main outcomes of the meta-analysis were proportions of cases that compared short-term (<5 years) complication rates between stemless and conventional stemmed shoulder implants. However, weighted mean difference (WMD) was calculated for postoperative CS and ROM because the same measurement tools were used to measure the same outcome. For all comparisons, odds ratios (ORs) and 95% confidence intervals (CIs) were calculated for binary outcomes, while WMDs and 95% CIs were calculated for continuous outcomes. When standard deviations (SDs) were not included in the original studies, they were calculated from the CIs or *P* values. Heterogeneity was determined by estimating the proportion of between-study inconsistencies due to actual differences between studies, rather than differences due to random error or chance. We assumed the presence of heterogeneity a priori and used the random-effects model in all pooled analyses. *I*^2^ statistics with a value less than 40% represent low heterogeneity, and a value of 75% or more indicates high heterogeneity.^[18]^ When statistical heterogeneity was substantial, we conducted metaregression to identify potential sources of bias such as study type and sample size. The age of the study subjects was also considered. All statistical analyses were performed with RevMan version 5.3 software and Stata version 14.2 static software. Subgroup analyses based on differences in study type and implant were performed for postoperative CS to explore a potential source of heterogeneity. As a result, 2 subgroups were created in each group: RCT (randomized control trial) or PCS (prospective comparative study) and RCS (retrospective comparative study) for (prospective comparative study) and TSA (total shoulder arthroplasty) and RSA (reverse shoulder arthroplasty) for implant. To detect the effect of individual studies on the pooled effect, sensitivity analysis was performed; only 1 study^[19]^ with a deltoid splitting approach were included. Pooling of data was feasible for 2 outcomes of interest: postoperative CS and complication rates.

## 3 Results

### 3.1 Study identification, study characteristics, patient populations, quality assessment, and publication bias of included studies

**Figure 1:**
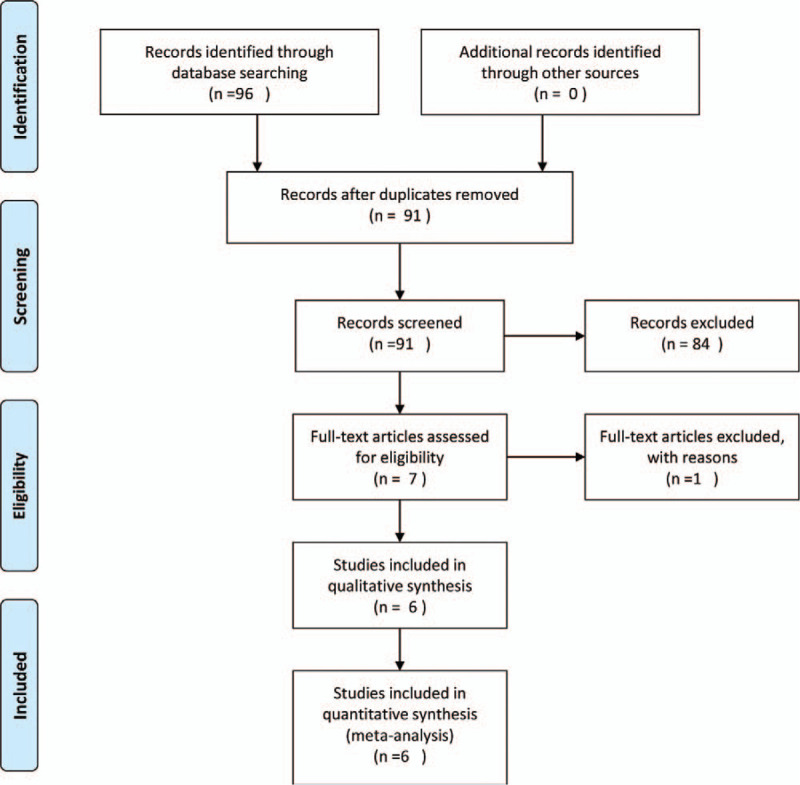
A flow diagram of preferred reporting items for systemic reviews and meta-analyses (PRISMA).

Details on study identification, inclusion, and exclusion are summarized in Figure 1. This process eventually resulted in 6 studies in the final meta-analysis.^[3,4,7,19–21]^ An electronic search yielded 581 studies in PubMed (MEDLINE), 778 in EMBASE, 619 in Web of Science, 763 in SCOPUS, and 24 in the Cochrane Library. Three additional publications were identified through manual searching. The 6 studies we examined comprised 122 subjects with stemless shoulder implants and 122 subjects with conventional stemmed shoulder implants that reported postoperative CS, ROM, or complication rates. Four studies (2 RCT and 2 PCS) compared prospectively measured parameters, whereas the other 2 studies compared parameters measured by retrospective chart review. Five studies compared groups according to postoperative CS, 5 compared groups according to ROM, and 6 compared groups according to complication rates (Table 1). The quality of the 6 studies is summarized in Table 1. Inter-rater reliability (k values) for all items of the Newcastle-Ottawa Scale ranged from 0.77 to 0.85, suggesting more than substantial agreement between the 2 investigators. Publication bias could not be assessed in these trials. Tests for funnel plot asymmetry are typically performed only when at least 10 studies are included in the meta-analysis.^[22]^ As our analysis included only 6 studies, tests for asymmetry were not performed because these tests would not be able to differentiate asymmetry from chance.

**Table 1:** Summary of patient characteristic of the included studies.

Study	Year	Study type	Prosthesis properties	Mean age (years)	Sample size	Follow-up (months)	Approach	Measured parameters
Uschok et al	2017	RCT	TSA Stemless (Eclipse stemless shoulder prosehesis, Arthrex Inc., Freiham, Germany)	65	15	68	DP	ROM, CS, radiologic finding
			TSA Stemmed (Univers II prosthesis, Arthrex Inc., Freiham, Germany)	69	18	70	DP	ROM, CS, radiologic finding
Moroder et al	2016	CCS	RSA Stemless (TESS, Biomet, Inc.,Warsaw, IN, USA)	76	24	34.2	DP	ROM, CS, radiologic finding
			RSA Stemmed (DELTA XTEND, Depuy Synthes Inc., Warsaw, IN, USA)	74	24	35.2	DP	ROM, CS, radiologic finding
Maier et al	2015	CCS	TSA Stemless (TESS, Biomet, Inc.,Warsaw, IN, USA)	68	12	6	DP	ROM, CS
			TSA Stemmed (Aequalis, Tounier, lyon, France)	68	12	6	DP	ROM, CS
Mariotti et al	2014	RCT	TSA Stemless (Aequalis stemless press-fit resurfacing head, Tornier Inc., France)	NA	9	24	DP	ROM, CS
			TSA Stemmed (Aequalis shoulder prosthesis system, Tornier Inc., France)	NA	10	24	DP	ROM, CS
Kadum et al.	2014	PCS	RSA Stemless (TESS, Biomet, Inc.,Warsaw, IN, USA)	69	16	35	AS	ROM,Quick DASH
			RSA Stemmed (TESS, Biomet, Inc.,Warsaw, IN, USA)	72	15	35	AS	ROM, Quick DASH
Berth et al	2013	PCS	TSA Stemless (TESS, Biomet Inc., warsaw, IN, USA)	67	41	31	DP	ROM, CS
			TSA Stemmed (Affinis, Mathys, Bettlach, Swittzerland)	67	41	33	DP	ROM, CS

AS = anterosuperior approach, CS = constant score, DP = deltopectoral approach, PCS = prospective comparative non-randomized study, RCS = retrospective comparative study, RCT = randomized controlled trial, RSA = reverse shoulder arthroplasty NA = not available, TSA = total shoulder arthroplasty.

### 3.2 Postoperative CS

**Figure 2:**
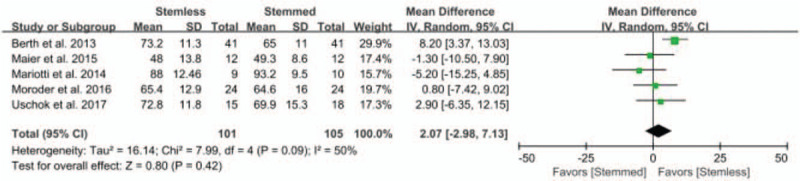
Results of aggregate analysis for comparison of postoperative Constant score (CS) between patients with stemless and conventional stemmed shoulder prosthesis. CS = constant score.

Of the 6 studies, 5 compared postoperative CS between patients with stemless shoulder implants (n = 202) and conventional stemmed shoulder implants (n = 210). The pooled data showed that the weighted mean postoperative CS was 2.27 points (95% CI: −1.04 to 5.57 points; *P* = .18; *I*^2^ = 44%, Fig. 2), with no significant difference between groups. Five studies were assigned to the study type (RCT or PCS and RCS) and implant (TSA and RSA) subgroups. The RCT or PCS subgroup with stemless shoulder implants was −0.90 points less than the RCT or PCS subgroup with conventional stemmed shoulder implants, although this difference was not significant (95% CI: −8.83 to 7.02 points; *P* = .82; *I*^2^ = 26%, Fig. 2). The RCS subgroup with conventional stemmed shoulder implants was 3.52 points less than the RCS subgroup with stemless shoulder implants, although this difference was not significant (95% CI: −2.75 to 9.80 points; *P* = .27; *I*^2^ = 55, Fig. 2). Similarly, the TSA and RSA subgroups with stemless shoulder implants showed 2.10 points greater (95% CI: −4.25 to 8.45 points; *P* = .52; *I*^2^ = 60%, Fig. 2) and 0.80 points greater (95% CI: −7.42 to 9.02 points; *P* = .85; *I*^2^ = not applicable, Fig. 2) values than the TSA and RSA subgroups with conventional stemmed shoulder implants, respectively.

### 3.3 Postoperative ROM

**Figure 3:**
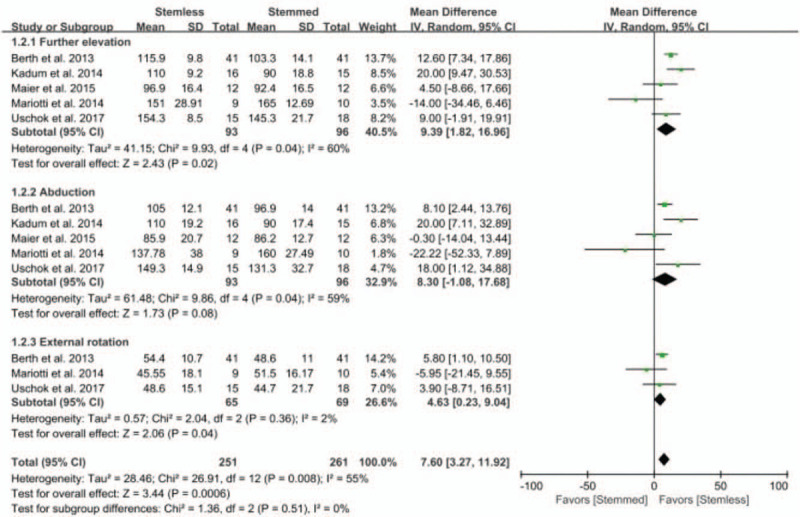
Results of aggregate analysis for comparison of postoperative range of motion (ROM) between patients with stemless and conventional stemmed shoulder prosthesis, including subgroup analysis by forward elevation, abduction, and external rotation. ROM = range of motion.

Of the 6 studies, 5 compared the range of shoulder motion between the 2 groups. The pooled mean difference in the range of shoulder motion was 7.60 degrees (95% CI: 3.27–11.92 degrees; *P* < .01; *I*^2^ = 55%, Fig. 3), with significant difference between the stemless and stemmed groups. Five studies were included in the forward elevation subgroups, 5 were included in the abduction subgroups, and 3 were included in the external rotation subgroups. For the forward elevation subgroup, the stemless shoulder implants led to 9.39 degrees greater forward elevation than the conventional stemmed shoulder implants, and this difference was significant (95% CI: 1.82–16.96 degrees; *P* = .02; *I*^2^ = 60%, Fig. 3). For the external rotation subgroup, the stemless shoulder implants led to 4.63 degrees greater external rotation than the conventional stemmed shoulder implants, and this difference was significant (95% CI: 0.23–9.04 degrees; *P* = .04; *I*^2^ = 2%, Fig. 3). In contrast, the pooled mean difference in the abduction subgroup was 8.30 degrees (95% CI: −1.08 to 17.68 degrees; *P* = .08; *I*^2^ = 59%, Fig. 3), indicating that abduction was not significantly greater for the stemless than the conventional stemmed shoulder implants. The sensitivity analysis found no significant differences compared to the original analysis (Table 2).

**Table 2:** Summary of mean difference for outcomes of subgroup analysis in terms of study type and implant.

Outcome or subgroup	Number of studies	Participants (Stemless/Stemmed)	ES (95% CI)	*I*^2^ (%)	*P* value
Constant score			MD		
All	5	101/105	2.07 (−2.98 to 7.13)	50	.42
Subgroup analysis					
Study type					
RCT or PCS	2	24/28	−0.90 (−8.83 to 7.02)	26	.82
RCS	3	77/77	3.52 (-2.75 to 9.80)	55	.27
Implant					
TSA	4	77/81	2.10 (−4.25 to 8.45)	60	.52
RSA	1	24/24	0.80 (−7.42 to 9.02)	NA	.85

CI = confidence interval, ES = effect size, MD = mean difference, NA = not available, PCS = prospective comparative study, RCS = retrospective comparative study, RCT = randomized control trial, RSA = reverse shoulder arthroplasty, TSA = total shoulder arthroplasty.

### 3.4 Complication rates

**Figure 4:**
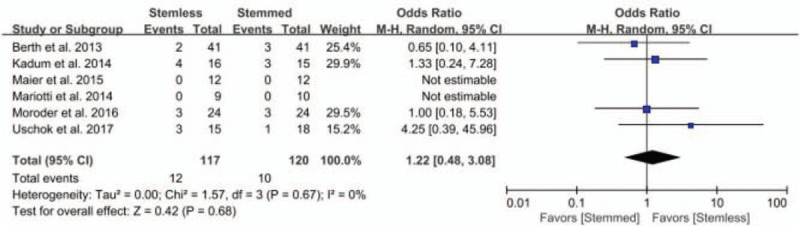
Results of aggregate analysis for comparison of complication rates between between patients with stemless and conventional stemmed shoulder prosthesis.

Six studies compared complication rates between groups (stemless shoulder implants, 12/117; conventional stemmed shoulder implants, 10/120; OR 1.22, 95% CI: 0.48 to 3.08; *P* = .68; *I*^2^ = 0%, Fig. 4). The sensitivity analysis found no significant differences compared to the original analysis, indicating that the findings were robust to decisions made in the data collection process (Table 3).

**Table 3:** Sensitivity analysis.

Study	Parameter	Before exclusion	After exclusion	Statistical significance
Kadum et al.^[3]^ (2014)	ROM	MD = 7.60, 95% CI = 3.27 to 11.92, Z = 3.44, *P* = .0006	MD = 5.79, 95% CI = 1.63 to 9.95, Z = 2.73, *P* = .006	No difference
	Complications	OR = 1.22, 95% CI = 0.48,3.08, Z = 0.42, *P* = .68	OR = 1.17, 95% CI = 0.39,3.55, Z = 0.28, *P* = .78	No difference

CI = confidence interval, CR = complication rate, MD = mean difference, OR = odd ratio, ROM = range of motion.

### 3.5 Meta-regression analysis

The results of the meta-regression analysis are summarized in Table 4. For complication rates of the stemless shoulder implants, sample size (*P* = .186), age (*P* = .462), and average follow-up (*P* = .547) were not significant sources of heterogeneity. Similarly, sample size (*P* = .995), age (*P* = .341), and average follow-up (*P* = .567) were not significant sources of heterogeneity for complication rates of the conventional stemmed shoulder implants.

**Table 4:** Meta-regression analyses of potential sources and difference in complication for stemless and stemmed implants.

Variable	Coefficient	Standard error	*P* value	95% confidence interval
Complication (Stemless)				
Number of patients (≤20 or ≥20)	−0.159	0.081	.186	−0.507 to 0.187
Age, mean, year (≤70 or ≥70)	0.083	0.091	.462	−0.313 to 0.478
Average follow-up (≤5years or ≥5years)	0.095	0.133	.547	−0.475 to 0.665
Complication (Stemmed)				
Number of patients (≤20 or ≥20)	0.000	0.059	.995	−0.254 to 0.255
Age, mean, year (≤70 or ≥70)	0.081	0.065	.341	−0.200 to 0.361
Average follow-up (≤5years or ≥5years)	-0.043	0.064	.567	−0.315 to 0.229

## 4 Discussion

This pairwise meta-analysis analyzed 6 studies comprising 122 subjects treated with stemless shoulder prosthesis and 122 subjects treated with stemmed shoulder prosthesis. The results indicated that postoperative CS and complication rates did not differ significantly between the 2 treatment methods for shoulder arthropathy. However, stemless shoulder prosthesis resulted in better outcomes than conventional stemmed shoulder prosthesis in terms of postoperative ROM.

There is still not a consensus on the exact pattern of postoperative CS after stemless or stemmed shoulder arthroplasty. In a previous short-term follow-up (32 months) study of 82 patients with primary OA of the shoulder treated with either stemless or stemmed prosthesis, stemless group was significantly lower estimated blood loss and mean operative time than stemmed group, but no significant difference in postoperative CS.^[7]^ This finding corresponds well with the results of a recent study reporting that postoperative CS improved significantly in both new stemless and fourth-generation standard stemmed groups, with no significant difference between the minimum of 2-year and 5-year follow-ups.^[4]^ Conversely, another study reported that in the stemless group, the CS improved significantly at a 6 month follow-up, even with relatively low postoperative CS caused by short follow-up period compared to other studies.^[3]^ In the current meta-analysis, postoperative CS showed no significant difference between the stemless and stemmed groups. Moreover, our subgroup analysis that evaluated mean difference for postoperative CS in different study type (RCT or PCS vs RCS) and implant design (TSA vs RTSA) suggested that no significant difference in postoperative CS between the groups according to study type and implant design.

Previous studies investigating ROM in patients treated with either stemless or stemmed prosthesis found no statistically significant difference in terms of overall active ROM between the implant groups^[4]^ and abduction or rotational motion; however, there was a trend toward the stemless reverse shoulder arthroplasty (RSA) having greater internal rotation than the stemmed RSA.^[21]^ Theoretically, preoperative ROM is the most important predictive risk factor of postoperative ROM in shoulder arthroplasty, and patients with higher preoperative ROM have higher postoperative ROM than patients with lower preoperative ROM after stemless or stemmed shoulder arthroplasty. Unlike postoperative CS, we observed that in the stemless group, postoperative forward elevation and external rotation were higher than its counterpart. As expected, this finding may have been due to higher preoperative forward elevation and external rotation values in the stemless group, suggesting that it was less likely to be attributable to the difference in implant even though we could not determine whether preoperative ROM was significantly different between the 2 groups owing to the limited data reported in the original papers.

Our meta-analysis also revealed no significant difference in the incidence of complications that required reoperation between the stemless and stemmed groups. Stemless shoulder arthroplasty should prevent further revision surgery by mitigating additional bone loss and show satisfactory outcomes in short- to midterm follow-up.^[3,4,19]^ These findings are similar to those of a recent study that no complication was related to the humeral components during a midterm follow-up period of 8 years, and the survival rate in the stemless TSA was comparable with that in the stemmed TSA. Moreover, they observed no complications related to the 12 cases of stemless humeral implantation after the 8.4-year follow-up of RSA.^[5]^ Considering the possible influence of number of patients, age, and average follow-up on researching performance, we further evaluated this issue by meta-regression analysis. For the stemless group, number of patients, age, and average follow-up did not appear to be the probable source of heterogeneity. The same was true of the stemmed group. This possibility is supported by the *I*^2^ results of complications, in that the current meta-analysis had no remarkable heterogeneity (*I*^2^ = 0%). In addition, excluding the possible influence of the surgical approach, we evaluated it with and without the article using deltoid splitting approach by sensitivity analysis. Unfortunately, we did not identify the source of approach heterogeneity in this meta-analysis. Together, these results suggest that, clinically, the stemless shoulder arthroplasty could be an effective surgical method to avoid stem loosening even though a longer follow-up is necessary and recommended to be used selectively to treat young patients without the problem of the proximal humerus metaphysis and poor bone quality.^[23]^

This study had several limitations. Of the 6 studies, 4 were observational, resulting in some inherent heterogeneity due to uncontrolled bias, even though the studies had high quality scores. In addition, the heterogeneity of the included studies could also be explained by slight differences in other factors affecting clinical outcomes, including the use of a wide variety of subject characteristics and prosthetic designs.

## 5 Conclusions

This pairwise meta-analysis revealed that constant score and complication rates did not differ significantly between the 2 treatment methods, stemless shoulder prosthesis and conventional stemmed shoulder prosthesis. However, stemless shoulder prosthesis resulted in better outcomes than conventional stemmed shoulder prosthesis in terms of postoperative ROM. Based on the findings of the current meta-analysis, stemless shoulder prosthesis with metaphyseal fixation appears to be as efficacious and safe as conventional stemmed shoulder prosthesis in the treatment of shoulder arthropathy even though long-term and high-quality randomized control trials are needed to confirm the clinical benefits of both the 2 techniques.

## Author contributions

**Conceptualization:** Young-Soo Shin, Woo-Seung Lee, Jun-Sung Won.

**Data curation:** Young-Soo Shin, Woo-Seung Lee, Jun-Sung Won.

**Formal analysis:** Young-Soo Shin, Woo-Seung Lee, Jun-Sung Won.

**Funding acquisition:** Woo-Seung Lee.

**Investigation:** Young-Soo Shin, Jun-Sung Won.

**Methodology:** Young-Soo Shin, Jun-Sung Won.

**Resources:** Jun-Sung Won.

**Software:** Jun-Sung Won.

**Supervision:** Woo-Seung Lee, Jun-Sung Won.

**Validation:** Young-Soo Shin, Woo-Seung Lee.

**Visualization:** Young-Soo Shin, Jun-Sung Won.

**Writing – original draft:** Young-Soo Shin, Jun-Sung Won.

**Writing – review & editing:** Young-Soo Shin, Woo-Seung Lee, Jun-Sung Won.

## Abbreviations

Abbreviations: AAOS = American Academy of Orthopedic Surgeons, CIs = confidence intervals, CS = constant score, ES = effect size, MD = mean difference, NA = not available, OARSI = Osteoarthritis Research Society International), ORs = odds ratios, PCS = prospective comparative study, RCS = retrospective comparative study), RCT = randomized control trial, ROM = range of motion, RSA = reverse shoulder arthroplasty, SDs = standard deviations, TSA = total shoulder arthroplasty, WMD = weighted mean difference.
